# Supplementary material for: Effects of environmental variables on the distribution of juvenile cubomedusae Carybdea marsupialis in the coastal Western Mediterranean
Source: PLoS One. 2020 Jun 17;15(6):e0230768. doi: 10.1371/journal.pone.0230768 (PMC7299366; doi:10.1371/journal.pone.0230768)
Supplement: S1 Table — From North to South: Río Racons (RR), Deveses (DEV), Almadrava Norte (AN), Almadrava Centro Norte (ACN), Almadrava Centro Sur (ACS), Almadrava Sur (AS), Molins (MO), Blay Beach (BB), Raset (RA), Marineta Casiana (MA) and Rotas (RO). (DOCX) [file pone.0230768.s010.docx]

**S1 Table.**

|  | January | | March | | May | | June | | July | | August | | October | | November | |
| --- | --- | --- | --- | --- | --- | --- | --- | --- | --- | --- | --- | --- | --- | --- | --- | --- |
|  | MEAN | SD | MEAN | SD | MEAN | SD | MEAN | SD | MEAN | SD | MEAN | SD | MEAN | SD | MEAN | SD |
| 1-RR | 14.33 | 0.13 | 14.78 | 0.44 | 20.18 | 0.19 | 23.87 | 1.24 | 28.00 | 0.27 | 27.38 | 0.37 | 21.14 | 0.16 | 19.67 | 0.08 |
| 2-DEV | 13.93 | 0.23 | 14.86 | 0.53 | 20.08 | 0.34 | 23.87 | 1.10 | 27.91 | 0.14 | 27.13 | 0.34 | 20.71 | 0.19 | 19.07 | 0.27 |
| 3-AN | 13.83 | 0.19 | 15.00 | 0.76 | 20.22 | 0.73 | 23.65 | 0.51 | 27.91 | 0.10 | 26.90 | 0.11 | 20.81 | 0.08 | 19.35 | 0.43 |
| 4-ACN | 14.01 | 0.16 | 14.50 | 0.42 | 20.34 | 0.64 | 23.95 | 0.42 | 27.80 | 0.28 | 27.05 | 0.10 | 20.90 | 0.09 | 19.16 | 0.19 |
| 5-ACS | 14.13 | 0.18 | 14.75 | 0.87 | 20.34 | 0.90 | 23.86 | 0.65 | 27.75 | 0.34 | 27.02 | 0.07 | 20.95 | 0.11 | 19.16 | 0.08 |
| 6-AS | 14.26 | 0.10 | 14.77 | 0.85 | 20.43 | 0.80 | 23.69 | 0.61 | 27.88 | 0.34 | 27.03 | 0.08 | 20.96 | 0.25 | 19.26 | 0.40 |
| 7-MO | 14.05 | 0.10 | 14.47 | 0.39 | 20.53 | 0.98 | 23.77 | 0.09 | 28.09 | 0.67 | 27.12 | 0.14 | 20.57 | 0.58 | 18.45 | 0.40 |
| 8-BB | 13.99 | 0.30 | 14.70 | 0.33 | 20.71 | 0.97 | 24.03 | 0.59 | 28.21 | 0.31 | 27.55 | 0.62 | 20.32 | 0.31 | 18.97 | 0.36 |
| 9-RA | 14.61 | 0.50 | 15.10 | 0.65 | 21.79 | 1.53 | 24.38 | 0.52 | 28.14 | 1.13 | 27.53 | 0.43 | 20.10 | 0.36 | 19.58 | 0.38 |
| 10-MA | 14.88 | 0.47 | 14.08 | 0.45 | 19.46 | 0.57 | 23.79 | 0.67 | 27.98 | 1.90 | 27.19 | 1.02 | 20.21 | 0.75 | 18.62 | 0.73 |
| 11-RO | 14.80 | 0.45 | 13.23 | 0.07 | 18.81 | 0.47 | 22.68 | 0.19 | 26.67 | 0.23 | 26.54 | 0.19 | 20.62 | 0.88 | 19.22 | 0.27 |
| ALL SITES | 14.26 | 0.44 | 14.56 | 0.72 | 20.26 | 1.02 | 23.77 | 0.74 | 27.85 | 0.77 | 27.12 | 0.46 | 20.66 | 0.49 | 19.14 | 0.48 |
