# Supplementary material for: Effects of environmental variables on the distribution of juvenile cubomedusae Carybdea marsupialis in the coastal Western Mediterranean
Source: PLoS One. 2020 Jun 17;15(6):e0230768. doi: 10.1371/journal.pone.0230768 (PMC7299366; doi:10.1371/journal.pone.0230768)
Supplement: S7 Table — (DOCX) [file pone.0230768.s016.docx]

**S7 Table.**

|  | Caryb | l.Temp | l.Sal | l.DIN | l.P | l.Si | l.Chla | Zoopl | Copep | Clado |
| --- | --- | --- | --- | --- | --- | --- | --- | --- | --- | --- |
| Caryb | 1 | 0.09 | 0.04 | -0.02 | -0.12 | 0.05 | -0.04 | -0.05 | -0.04 | -0.05 |
| l.Temp | 0.09 | 1 | 0.05 | 0.14 | 0.02 | 0.21 | 0.13 | 0.14 | 0.10 | 0.22 |
| l.Sal | 0.04 | 0.05 | 1 | -0.38 | -0.46 | -0.61 | -0.31 | 0.03 | 0.01 | 0.05 |
| l.DIN | -0.02 | 0.14 | -0.38 | 1 | 0.30 | 0.42 | 0.42 | 0.16 | 0.16 | 0.04 |
| l.P | -0.12 | 0.02 | -0.46 | 0.30 | 1 | 0.34 | 0.38 | -0.07 | 0.01 | 0.03 |
| l.Si | 0.05 | 0.21 | -0.61 | 0.42 | 0.34 | 1 | 0.41 | 0.06 | 0.11 | 0.04 |
| l.Chla | -0.04 | 0.13 | -0.31 | 0.42 | 0.38 | 0.41 | 1 | 0.05 | 0.05 | 0.12 |
| Zoopl | -0.05 | 0.14 | 0.03 | 0.16 | -0.07 | 0.06 | 0.05 | 1 | 0.95 | 0.44 |
| Copep | -0.04 | 0.10 | 0.01 | 0.16 | 0.01 | 0.11 | 0.05 | 0.95 | 1 | 0.37 |
| Clado | -0.05 | 0.22 | 0.05 | 0.04 | 0.03 | 0.04 | 0.12 | 0.44 | 0.37 | 1 |
